# Vitamin D and the Epithelial to Mesenchymal Transition

**DOI:** 10.1155/2016/6213872

**Published:** 2016-01-06

**Authors:** María Jesús Larriba, Antonio García de Herreros, Alberto Muñoz

**Affiliations:** ^1^Instituto de Investigaciones Biomédicas “Alberto Sols”, Consejo Superior de Investigaciones Científicas, Universidad Autónoma de Madrid, IdiPAZ, 28029 Madrid, Spain; ^2^Institut Hospital del Mar d'Investigacions Mèdiques, 08003 Barcelona, Spain; ^3^Departament de Ciències Experimentals i de la Salut, Universitat Pompeu Fabra, 08003 Barcelona, Spain

## Abstract

Several studies support reciprocal regulation between the active vitamin D derivative 1*α*,25-dihydroxyvitamin D_3_ (1,25(OH)_2_D_3_) and the epithelial to mesenchymal transition (EMT). Thus, 1,25(OH)_2_D_3_ inhibits EMT* via* the induction of a variety of target genes that encode cell adhesion and polarity proteins responsible for the epithelial phenotype and through the repression of key EMT inducers. Both direct and indirect regulatory mechanisms mediate these effects. Conversely, certain master EMT inducers inhibit 1,25(OH)_2_D_3_ action by repressing the transcription of *VDR* gene encoding the high affinity vitamin D receptor that mediates 1,25(OH)_2_D_3_ effects. Consequently, the balance between the strength of 1,25(OH)_2_D_3_ signaling and the induction of EMT defines the cellular phenotype in each context. Here we review the current understanding of the genes and mechanisms involved in the interplay between 1,25(OH)_2_D_3_ and EMT.

## 1. The Vitamin D System

The mammalian form of vitamin D is the prohormone vitamin D_3_ (cholecalciferol), which is obtained from the diet or mainly synthesized in the skin from 7-dehydrocholesterol through ultraviolet B radiation. Vitamin D_3_ is hydroxylated, first in the liver and then in the kidney and other tissues, to generate 1*α*,25-dihydroxyvitamin D_3_ (1,25(OH)_2_D_3_, calcitriol), the most active vitamin D_3_ metabolite [1–5]. 1,25(OH)_2_D_3_ is a major regulator of gene expression and exerts its effects by binding to a transcription factor of the nuclear receptor superfamily: the vitamin D receptor (VDR). VDR heterodimerizes with another member of the same family, the retinoid X receptor, and regulates gene expression in a ligand-dependent manner. The prevailing model holds that in the absence of 1,25(OH)_2_D_3_ the heterodimer is bound to specific sequences on its target genes (vitamin D response elements) and to transcriptional corepressors that recruit complexes with histone deacetylase activity, thus maintaining the chromatin in a transcriptionally repressed state. 1,25(OH)_2_D_3_ induces conformational changes in VDR that cause the release of corepressors and the binding of coactivators and chromatin remodelers. Together they mediate chromatin opening and permit the entry of the basal RNA polymerase II transcription machinery and transcription initiation [1, 6–9].

1,25(OH)_2_D_3_ is a pleiotropic hormone with many regulatory effects. It was classically known for its action on calcium and phosphorus homeostasis and bone mineralization [2, 10]. The seminal discoveries in 1981 that 1,25(OH)_2_D_3_ induced myeloid leukemia cell differentiation and inhibited melanoma cell proliferation prompted the interest in 1,25(OH)_2_D_3_ as an anticancer agent [11, 12]. Subsequent observations have shown that 1,25(OH)_2_D_3_ induces differentiation and apoptosis and inhibits proliferation, migration, invasion, and angiogenesis in cancer cells of different origin and in several animal models of cancer [1, 5, 13–16]. However, the administration of 1,25(OH)_2_D_3_ to cancer patients is restricted by its hypercalcemic effects at the therapeutic doses, enforcing the development of several analogs that maintain the antitumoral properties but have less calcemic actions. Currently, numerous clinical trials are ongoing using 1,25(OH)_2_D_3_ or its analogs, alone or in combination with other anticancer agents, against several neoplasms (https://www.clinicaltrials.gov/) [1, 4, 5, 13].

## 2. Epithelial to Mesenchymal Transition

Epithelial to mesenchymal transition (EMT) is the process by which epithelial cells are converted into mesenchymal cells. It takes place physiologically in several developmental situations such as mesoderm formation and neural crest migration. In the adult, it is reactivated in certain pathological conditions such as wound healing, fibrosis, and cancer progression [17, 18]. During EMT, epithelial cells lose cell-cell and cell-extracellular matrix junctions, change from an apical-basal to a front-rear polarity, reorganize their cytoskeleton, and undergo a gene expression reprogramming characterized by the downregulation of the epithelial gene signature and the activation of mesenchymal genes. This process generates motile individual cells that can degrade the extracellular matrix and thus develop a migratory and invasive phenotype [18–21]. EMT is a highly regulated, plastic, and reversible process. Thus, the mesenchymal to epithelial transition (MET) occurs under certain conditions and enables that mesenchymal cells acquire an epithelial state [22–25].

Typical EMT gene reprogramming is mainly orchestrated by key transcription factors including the zinc finger proteins SNAIL1 and SNAIL2, the double zinc finger and homeodomain factors ZEB1 and ZEB2, and the members of the basic-helix-loop-helix family TWIST1 and E47, all known as EMT transcription factors (EMT-TFs). They are repressors of E-cadherin (encoded by* CDH1*), that is the main component of adherens junctions and essential for the maintenance of the epithelial state. Thus, E-cadherin downregulation is considered a hallmark of EMT. In addition to the established EMT inducers, other transcription factors such as FOXC2, Goosecoid, KLF8, TCF4 (also known as E2-2), SIX1, HMGA2, Brachyury, and PRRX1 have been recently shown to induce or regulate EMT [18–21, 24]. Expression and/or activity of the transcription factors that drive EMT is induced and controlled by several signaling pathways that respond to extracellular cues, with a prominent role for transforming growth factor- (TGF-) *β* signaling. The contribution of each transcription factor to the EMT depends on the cell or tissue type involved and the signaling pathway that initiates the EMT. Moreover, EMT-TFs often exhibit reciprocal control of their expressions and functional cooperation [18–20, 22, 23].

## 3. 1,25(OH)_2_D_3_ Inhibits EMT

### 3.1. 1,25(OH)_2_D_3_ Induces the Expression of Epithelial Markers

1,25(OH)_2_D_3_ induces epithelial differentiation in several normal and cancer cells. Accordingly, it increases the expression of components of almost all types of cell adhesion structures that are essential for the acquisition and maintenance of the epithelial phenotype (Table 1). Remarkably, we found that the strong prodifferentiation effect of 1,25(OH)_2_D_3_ in human colon cancer cells is associated with an increase in the expression of the key adhesion molecule E-cadherin. This is accompanied by the redistribution of *β*-catenin from the cell nucleus to the adherens junctions at the plasma membrane where it interacts with E-cadherin, thus inhibiting the Wnt/*β*-catenin signaling pathway that is aberrantly activated in most colon tumors and required for colon carcinogenesis [26]. In human colon cancer cells, 1,25(OH)_2_D_3_ also induces the expression of the tight junction components occludin, claudin-1, claudin-2, claudin-7, claudin-12,* zonula occludens*- (ZO-) 1 and ZO-2, the desmosomal protein plectin, the focal adhesion members integrin *α*_3_ and paxillin, the constituent of intermediate filaments keratin-13, and proteins associated with the actin cytoskeleton such as vinculin, filamin A, and ezrin [26, 27, 29–31, 49, 50]. Interestingly, 1,25(OH)_2_D_3_ downregulates cadherin-17 [72], which induces cell proliferation and has protumoral and prometastatic effects in colon cancer cells [73].

Treatment of the *Apc* ^min/+^ colon cancer mouse model with 1,25(OH)_2_D_3_ or analogs reduces polyp number and load, while it increases E-cadherin levels and reduces *β*-catenin nuclear localization and the expression of the *β*-catenin target genes* Tcf1*,* Myc*, and* Cd44* in the small intestine and colon [32]. Conversely, *Vdr* deficiency in *Apc* ^min/+^ mice enhances tumor size and the activity of the Wnt/*β*-catenin pathway in the lesions [65, 74]. Similar results were observed in other mouse and rat models of colon dysplasia, colon cancer, and colitis-associated neoplasia when treated with vitamin D_3_ or 1,25(OH)_2_D_3_ analogs [33, 63, 64]. Moreover, 1,25(OH)_2_D_3_ increases and restores the normal level of Zo-1, occludin, and claudin-1 proteins in the colonic epithelium of the dextran sulfate sodium- (DSS-) induced colitis mouse model, protecting mice from intestinal mucosa injury and epithelial barrier disruption [27]. Conversely, *Vdr* deficiency potentiates DSS effects in this model, as DSS-treated *Vdr* ^−/−^ mice have severely disrupted and opened tight junctions and desmosomes in the colonic epithelium and develop more severe colitis than wild-type animals [29]. These data indicate that 1,25(OH)_2_D_3_ contributes to the homeostasis and healing capacity of the colonic epithelium by preserving the stability and structural integrity of tight junctions [75]. Remarkably, a randomized, double-blind, placebo-controlled clinical trial showed that daily treatment of colorectal adenoma patients with 800 IU of vitamin D_3_ for 6 months increases E-cadherin expression in normal-appearing rectal mucosa [34, 76].

In addition to colon cancer, E-cadherin is induced by 1,25(OH)_2_D_3_ or analogs in normal mammary and bronchial epithelial cells and in tumor cell lines derived from breast, prostate, non-small cell lung, and squamous cell carcinomas, usually associated with an increase in epithelial differentiation, a reduction in cell migration and invasion, and the inhibition of Wnt/*β*-catenin signaling [35–41, 61, 77, 78]. We have described that the mechanism of E-cadherin induction by 1,25(OH)_2_D_3_ in human colon cancer cells is transcriptional indirect and requires the transient activation of the RhoA-ROCK-p38MAPK-MSK1 signaling pathway [26, 31]. Phosphatidylinositol 5-phosphate 4-kinase type II *β* is also needed for E-cadherin induction by 1,25(OH)_2_D_3_ in colon cancer cells [79]. In agreement with the transcriptional regulation, Lopes et al. showed that 1,25(OH)_2_D_3_ treatment causes partial demethylation of CpG sites of* CDH1* promoter in MDA-MB-231 triple-negative breast cancer cells [78]. Moreover, protein kinase C inhibitors block E-cadherin, P-cadherin, *α*-catenin, and vinculin translocation to cell-cell contacts and the assembly of adherens junctions promoted by 1,25(OH)_2_D_3_ in cultured human keratinocytes [80].

We reported that 1,25(OH)_2_D_3_ induces cell adhesion, inhibits cell migration and invasion, and profoundly affects the phenotype of human breast cancer cells [37]. It promotes the formation of focal adhesions by increasing the expression of integrin *α*_V_, integrin *β*_5_, paxillin, and focal adhesion kinase (FAK) proteins and also by inducing FAK phosphorylation. Additionally, 1,25(OH)_2_D_3_ reduces the expression of the mesenchymal marker N-cadherin and the myoepithelial proteins P-cadherin, integrin *α*_6_, integrin *β*_4_, and *α*-smooth muscle actin (*α*-SMA). Thus, 1,25(OH)_2_D_3_ reverts the myoepithelial features that are associated with more aggressive and lethal forms of human breast cancer [37]. Likewise, N-cadherin expression is strongly suppressed by 1,25(OH)_2_D_3_ in mouse osteoblast-like cells [47]. In line with these data, 1,25(OH)_2_D_3_ treatment blocks the EMT-associated cadherin switch (from E-cadherin to N-cadherin) in pancreatic cancer cells [42]. Notably, 1,25(OH)_2_D_3_ enhances corneal epithelial barrier function as corneal epithelial cells treated with 1,25(OH)_2_D_3_ show increased occludin levels, reduced permeability, and elevated transepithelial resistance, a measure of the functional integrity of tight junctions [28].

### 3.2. 1,25(OH)_2_D_3_ Inhibits the Expression of EMT-TFs

We and others have reported that 1,25(OH)_2_D_3_ regulates the expression of certain transcription factors that induce EMT and of several modulators of the epithelial phenotype that can influence the expression of the EMT inducers (Table 1). 1,25(OH)_2_D_3_ increases by a transcriptional indirect mechanism the expression of Jumonji Domain Containing 3 (JMJD3), a histone H3 lysine 27 demethylase with putative tumor suppressor activity. JMJD3 mediates the induction of a highly adhesive epithelial phenotype, the antiproliferative effect, the gene regulatory action, and the antagonism of the Wnt/*β*-catenin pathway promoted by 1,25(OH)_2_D_3_ in human colon cancer cells [66]. Moreover,* JMJD3* depletion upregulates SNAIL1, ZEB1, and ZEB2, increases the expression of the mesenchymal markers fibronectin and LEF1, and downregulates the epithelial proteins E-cadherin, claudin-1, and claudin-7. Accordingly,* JMJD3* and* SNAIL1* RNA expression correlate inversely in samples from human colon cancer patients [66]. The induction of ZEB1 by* JMJD3* depletion is associated with the downregulation of* miR-200b* and* miR-200c*, two microRNAs that target* ZEB1* RNA and inhibit ZEB1 protein expression [81].

1,25(OH)_2_D_3_ directly induces the expression of cystatin D, an inhibitor of cysteine proteases of the cathepsin family encoded by* CST5* gene. The binding of VDR to the* CST5* promoter induced by 1,25(OH)_2_D_3_ is accompanied by the release of the NCOR2 corepressor and an increase in histone H4 acetylation [67]. We found that cystatin D mediates the antiproliferative and prodifferentiation action of 1,25(OH)_2_D_3_ in human colon cancer cells. In addition, ectopic cystatin D expression inhibits proliferation, migration, anchorage-independent growth, and the Wnt/*β*-catenin pathway in cultured colon cancer cells and reduces tumor development in xenografted mice [67]. Cystatin D represses SNAIL1, SNAIL2, ZEB1, and ZEB2, whereas it induces the expression of E-cadherin and other adhesion proteins such as occludin and p120-catenin. Accordingly, cystatin D and E-cadherin protein expression directly correlate in human colorectal cancer, and loss of cystatin D is associated with poor tumor differentiation [67]. Notably, transcriptomic and proteomic studies comparing cystatin D-overexpressing and mock-transfected human colon cancer cells indicated that “cell adhesion, cell junction, and cytoskeleton” is one of the gene categories that englobes more cystatin D-regulated genes and proteins [82]. Remarkably, Swami et al. showed that the expression of cathepsin L, whose activity is inhibited by cystatin D, is downregulated by 1,25(OH)_2_D_3_ in breast cancer cells [68], while Zhang et al. described that silencing of cathepsin L suppresses the cell invasion and migration, the actin cytoskeleton remodeling, and the increase in SNAIL1 expression associated with TGF-*β*-promoted EMT in breast and lung cancer cells [83].

1,25(OH)_2_D_3_ reduces the expression of Sprouty-2, an intracellular modulator of growth factor tyrosine kinase receptor signaling involved in the regulation of cell growth, migration, and angiogenesis [69]. Sprouty-2 strongly inhibits the induction of intercellular adhesion and E-cadherin protein expression promoted by 1,25(OH)_2_D_3_, and gain- and loss-of-function experiments indicate that Sprouty-2 and E-cadherin repress each other in colon cancer cells. Accordingly, the protein expression levels of Sprouty-2 and E-cadherin correlate inversely in cultured and xenografted colon cancer cells and in biopsies from human colon cancer patients. In line with this, we found that Sprouty-2 induces ZEB1 expression without affecting ZEB2, SNAIL1, or SNAIL2 levels [69]. ZEB1 upregulation by Sprouty-2 results from the induction of the transcription factor ETS1 and the repression of several microRNAs (*miR-200* family and* miR-150*) that target* ZEB1* RNA. Through ZEB1 upregulation, Sprouty-2 represses E-cadherin, claudin-7, occludin, the tight junction modulator matriptase, the cell adhesion molecule EPCAM, and the epithelial splicing regulatory protein ESRP1 that inhibits EMT [84]. Taken together, these data point to Sprouty-2 as a potent inhibitor of the epithelial phenotype that is downregulated by 1,25(OH)_2_D_3_ in colon carcinoma cells.

Recently, effects of 1,25(OH)_2_D_3_ on the expression of several EMT-TFs have been described. 1,25(OH)_2_D_3_ inhibits SNAIL1 and ZEB1 expression in non-small cell lung carcinoma cells, accompanied by an increase in E-cadherin expression, vimentin downregulation, maintenance of the epithelial morphology, and inhibition of cell migration [40]. The low calcemic 1,25(OH)_2_D_3_ analog MART-10 inhibits EMT and cell migration and invasion in breast and pancreatic cancer cells through the downregulation of SNAIL1 and SNAIL2. In addition, MART-10 inhibits TWIST1 expression in breast cancer cells [42, 61]. Accordingly, Findlay et al. reported the inhibition of SNAIL1 and SNAIL2 by 1,25(OH)_2_D_3_ in human colon cancer cells [62]. Kaler et al. found that colon cancer cells stimulate tumor-associated macrophages to secrete interleukin- (IL-) 1*β*, which in turn promotes Wnt/*β*-catenin signaling, stabilizes SNAIL1 protein, and confers resistance to TRAIL-induced apoptosis in colon cancer cells [71]. They also found that 1,25(OH)_2_D_3_, by inhibiting the release of IL-1*β* by macrophages, downregulates SNAIL1 protein expression in colon cancer cells [71]. Similarly, Zhang et al. showed that tumor-associated macrophages induce EMT in breast cancer cells and that high VDR expression in cancer cells abrogates the macrophage-promoted E-cadherin loss, *α*-SMA upregulation, and increase in cell migration and invasion [43]. Furthermore, 1,25(OH)_2_D_3_ attenuates the enhancing effect of TGF-*β*1 on cell motility and on SNAIL1, N-cadherin, and vimentin expression in human bronchial epithelial cells [41] and inhibits the TGF-*β*1-stimulated EMT in rat lung epithelial cells [51].

Matrix metalloproteases (MMPs) are a family of zinc-dependent proteases that degrade components of the extracellular matrix and basement membrane. MMPs are regulated by the action of specific inhibitors: the tissue inhibitors of metalloproteases (TIMPs). Increased MMP activity is often associated with the EMT and confers invasive properties to cancer cells. Consistently with its inhibitory effect on EMT, 1,25(OH)_2_D_3_ downregulates the secretion of MMP2, MMP9, and MMP13 in prostate, breast, pancreatic, and squamous cell carcinoma cells and increases TIMP1 and TIMP2 activity in prostate and breast cancer cells [39, 42, 48, 59–61]. In addition, 1,25(OH)_2_D_3_ reduces the increase in MMP2 and MMP9 induced by TGF-*β*1 in human bronchial epithelial cells [41]. Through these mechanisms, 1,25(OH)_2_D_3_ inhibits the capacity of cancer cells to degrade the extracellular matrix and invade the surrounding tissue and may thus reduce tumor cell metastatic potential. Remarkably, several studies from Pérez-Fernández's group have demonstrated that 1,25(OH)_2_D_3_ represses the expression of the gene encoding the pituitary transcription factor 1 (PIT1) in breast cancer cells and that* PIT1* silencing downregulates SNAIL1, MMP1, and MMP13 proteins [70, 85, 86]. In agreement with this, high PIT1 protein expression correlates with elevated MMP1 and MMP13 levels, SNAIL1 protein expression, and presence of distant metastasis in invasive ductal breast carcinoma [85, 86].

Recent studies have established a link between the induction of EMT and the acquisition by epithelial cells of molecular and functional traits of stem cells. As stem cells can both self-renew and differentiate, these stemness-related properties confer tumor-initiating capacities to carcinoma cells that could be crucial for cancer cell survival during dissemination and for the establishment by the disseminated cancer cells of metastatic foci at anatomically distant sites [19–25]. Interestingly, Pervin et al. found that manipulation of VDR levels modulates the expression of key EMT-related proteins and dictates the stem cell characteristics of breast cancer cells. Thus, *VDR* overexpression in these cells upregulates E-cadherin, downregulates SNAIL1, TWIST1, and MMP9, and reduces cell ability to form mammospheres, an attribute of breast normal and cancer stem cells. Conversely, *VDR* silencing has the opposite effect [87].

### 3.3. 1,25(OH)_2_D_3_ Inhibits Fibrosis

In addition to cancer progression, EMT is reactivated in adult life during other pathological processes such as organ fibrosis. This process occurs in certain epithelial tissues after trauma or inflammatory injury and is characterized by excessive deposition of extracellular matrix and increased fibrous connective tissue. In this context, the EMT is part of the repair program and originates fibroblasts and other related cells for tissue regeneration. However, the disease usually progresses and the organ is finally composed mainly of activated fibroblasts and extracellular matrix, which may eventually lead to organ failure. The EMT inducer TGF-*β* is also involved in the fibrotic process [17, 23, 88]. Several studies from Liu's group showed that vitamin D compounds attenuate renal interstitial fibrosis by inhibiting EMT in tubular epithelial cells. These compounds decrease collagen and fibronectin deposition, downregulate the expression of SNAIL1, *α*-Sma, *β*-catenin, TGF-*β*1, and its type I receptor, reduce *β*-catenin nuclear localization, and preserve Vdr and E-cadherin levels in kidneys from an obstructive nephropathy mouse model that develops interstitial fibrosis. Moreover, treatment with vitamin D compounds or *VDR* overexpression in human renal proximal tubular epithelial cells abolishes the EMT promoted by TGF-*β*1, while *VDR* silencing has a sensitizing effect [44, 45, 89]. Interestingly, combination of the 1,25(OH)_2_D_3_ analog paricalcitol with trandolapril, an inhibitor of the angiotensin-converting enzyme used as standard treatment for chronic kidney disease, leads to additive reduction of renal fibrosis in the obstructive nephropathy mouse model [90]. Additionally, Nolan et al. indicated that paricalcitol inhibits TGF-*β*1-induced tubular EMT also under the hypoxic conditions commonly associated with chronic kidney disease [46], and Kim et al. reported that it attenuates the tubular EMT exogenously induced by 4-hydroxy-2-hexenal, an aldehyde product of lipid peroxidation [52].

Beneficial effects of 1,25(OH)_2_D_3_ have also been reported in liver fibrosis. Hepatic stellate cells play a central role in liver fibrosis as upon injury-induced activation they proliferate and secrete many extracellular matrix components. Vitamin D compounds reduce extracellular matrix deposition and ameliorate liver fibrosis in rat and mouse models [55, 56]. In addition, 1,25(OH)_2_D_3_ suppresses cell proliferation and downregulates cyclin D1 and *α*_1_ type I collagen expression in cultured hepatic stellate cells [55]. Potter et al. reported that the downregulation of *α*_1_ type I collagen by 1,25(OH)_2_D_3_ is mediated by VDR binding to a proximal Sp1 site and a distal vitamin D response element in the human* COL1A1* gene promoter [57]. Notably, a study from Ding et al. revealed that ligand-activated VDR antagonizes TGF-*β*1-dependent transcription of profibrotic genes in hepatic stellate cells [56]. TGF-*β*1 changes ligand activated-VDR binding sites in the genome promoting VDR binding to SMAD3 sites in the regulatory regions of profibrotic genes, which decreases SMAD3 occupancy at these sites causing transcriptional silencing of the genes and inhibiting fibrosis [56]. Thus, VDR ligands limit fibrosis by modulating the tissue response to TGF-*β*1. Ito et al. described that a similar mechanism takes place in renal fibrosis and showed that the C-terminal *α*-helix 12 of the ligand-binding domain of VDR is necessary for the interaction with SMAD3 and the suppression of TGF-*β* pathway [53]. Furthermore, they designed VDR ligands that selectively inhibit TGF-*β* signaling without activating VDR-mediated transcription and significantly attenuate renal fibrosis in mice without hypercalcemic effects [53]. Another mechanism involved in the inhibition of TGF-*β* pathway by vitamin D analogs in renal fibrosis has been described: maxacalcitol blocks the autoinduction of TGF-*β*1 expression through the recruitment of a complex between VDR and the SMAD3 phosphatase PPM1A to the* TGFB1* promoter, causing SMAD3 dephosphorylation and release from the promoter and, consequently, attenuating* TGFB1* gene expression [54].

Peritoneal dialysis induces changes in mesothelial cells that are reminiscent of those occurring during EMT, and that may finally lead to the development of fibrosis. Vitamin D compounds prevent the progression of peritoneal fibrosis in mouse and rat models and inhibit the TGF-*β*1-induced EMT-like process in human peritoneal mesothelial cells [58, 91]. Thus, a large body of evidence indicates that vitamin D compounds protect against organ fibrosis in different tissues by inhibiting EMT and/or TGF-*β* profibrotic action.

## 4. The Transcription Factors SNAIL1 and SNAIL2 Repress* VDR* Gene Expression and Inhibit 1,25(OH)_2_D_3_ Action

Cell responsiveness to 1,25(OH)_2_D_3_ mainly relays on VDR expression levels. VDR protein is expressed in almost all normal human cell types and tissues, and also in cancer cell lines and tumors of several origins [8, 92]. Remarkably, elevated VDR expression is associated with high tumor differentiation, absence of node involvement, and good prognosis in colon cancer [93–95], with lower tumor grade, late development of lymph node metastases, and longer disease-free survival in breast cancer [43, 96–98], and with improved overall survival in prostate and non-small cell lung cancer and melanoma [99–101]. However, certain cancer cell lines do not express VDR and are unresponsive to 1,25(OH)_2_D_3_. Accordingly, VDR downregulation has been observed in a proportion of melanomas and colon, breast, lung, and ovarian tumors [43, 94, 99, 102–104], which may jeopardize the response to therapy with vitamin D, 1,25(OH)_2_D_3_, or its analogs.

These lines of evidence prompted us to study the mechanisms responsible for VDR downregulation in cancer. We found that SNAIL1 represses the expression of VDR by binding to three E-boxes in the human *VDR* gene promoter. Moreover, SNAIL1 reduces *VDR* RNA half-life [105]. As a result, SNAIL1 overexpression in human colon cancer cells blocks the induction of E-cadherin expression and the acquisition of an epithelial phenotype promoted by 1,25(OH)_2_D_3_. Consequently, *β*-catenin is not relocated from the nucleus to the plasma membrane adherens junctions and the Wnt/*β*-catenin signaling remains active. SNAIL1 also abrogates the inhibitory effect of 1,25(OH)_2_D_3_ on cell proliferation and migration in cultured cells and the antitumoral action of the 1,25(OH)_2_D_3_ analog EB1089 in xenografted mice [105, 106]. Consistently, Knackstedt et al. have shown that the downregulation of *Vdr* observed in the colon of DSS-induced colitis mouse model is associated with an increase in the expression of SNAIL1 and its upstream regulator tumor necrosis factor- (Tnf-) *α* [107].

In addition to SNAIL1, we reported that its family member SNAIL2 represses *VDR* gene expression through the same E-boxes in the human *VDR* gene promoter and blocks the induction of an epithelial phenotype by 1,25(OH)_2_D_3_ in human colon cancer cells. Moreover, SNAIL1 and SNAIL2 show an additive repressive effect on *VDR* gene promoter [108]. Remarkably,* SNAIL1* and/or* SNAIL2* RNA upregulation was detected in 76% of colon tumors and significantly correlated with diminished *VDR* RNA expression. Indeed, the lowest *VDR* RNA levels were observed in those colon tumors that overexpress both EMT-TFs [95, 105, 108]. We also showed that* SNAIL1* RNA overexpression in colon tumors diminishes *VDR* RNA expression in the histologically normal tissue adjacent to the tumor, suggesting that SNAIL1-expressing colon cancer cells secrete signals that modulate VDR expression in neighboring cells [109].

The repression of *VDR* gene by SNAIL factors is not exclusive to colon cancer. It has been shown that SNAIL1 and SNAIL2 downregulate *VDR* gene expression and abrogate the antitumoral action of 1,25(OH)_2_D_3_ in human osteosarcoma and breast cancer cells [110, 111]. The two proximal E-boxes of the human *VDR* gene promoter are conserved in rat and mouse, while the most distal box is only partially conserved with one base substitution. Bai et al. found SNAIL1 binding only to the most proximal E-box of the rat *Vdr* promoter accompanied by deacetylation of histone H3 in samples from rat intestine and kidney. Accordingly, an inverse correlation between SNAIL1 and *Vdr* levels was observed in those tissues [112]. de Frutos et al. showed that the sustained activation of SNAIL1 in transgenic mice represses *Vdr* gene expression in osteoblasts. This downregulation blocks the Vdr-mediated induction of the osteoclast differentiation factor Rankl and inhibition of osteoprotegerin, a decoy Rankl receptor that inhibits osteoclastogenesis. Thus, *Vdr* gene downregulation by SNAIL1 in osteoblasts reduces the osteoclast population due to an impaired osteoclastogenesis. In addition, chromatin immunoprecipitation assays indicated that *Vdr* gene repression in mouse osteoblasts is mediated by SNAIL1 binding to the two proximal E-boxes of murine *Vdr* promoter [113].

VDR downregulation takes place also and contributes to E-cadherin loss during the EMT promoted by the proinflammatory cytokine TNFSF12 in renal tubular epithelial cells [114]. Similarly, VDR repression by TNF-*α* sensitizes breast cancer cells to TGF-*β*1-induced EMT. Of note, 1,25(OH)_2_D_3_ treatment protects against TNF-*α*-induced VDR loss, suppresses TGF-*β*1-promoted increase in the migration capacity of cultured breast cancer cells, and inhibits lung metastasis in an orthotopic breast cancer mouse model [43]. Conversely, the MET induced by the enforced reexpression of the putative tumor suppressor KLF4 in hepatocellular carcinoma cells was accompanied by VDR upregulation and an increase in the inhibitory effect of 1,25(OH)_2_D_3_ on cell proliferation. As a result, KLF4 and VDR protein expression correlate directly in human hepatocellular carcinoma [115].

Other EMT-TFs such as ZEB1, ZEB2, TWIST1, or E47 have no effect on the expression of human *VDR* gene promoter in SW480-ADH human colon cancer cells [108]. However, Lazarova et al. reported that ZEB1 binds to two distal E-boxes in the murine *Vdr* promoter and activates its expression in COS-7 monkey kidney fibroblasts and SW620 human colon cancer cells, but not in human LNCaP prostate or HCT116 colon cancer cells [116]. Other studies showed absence of correlation or a significant direct correlation between* ZEB1* and *VDR* RNA expression in colon cancer [95, 109, 117]. Furthermore, Peña et al. observed that such direct correlation was stronger in colon tumors with high level of the transcriptional coactivator p300 [117]. Globally, these data suggest a cell- and context-dependent positive regulation of VDR by ZEB1.

## 5. Conclusions and Perspectives

**Figure 1:**
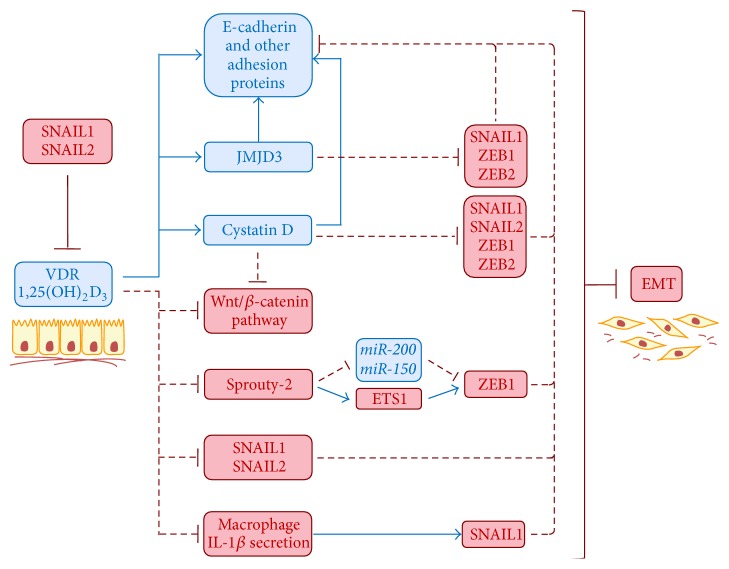
Scheme showing the mechanisms involved in the reciprocal regulation between 1,25(OH)_2_D_3_ and EMT in human colon cancer cells. Proteins and pathways displayed in blue are associated with an epithelial phenotype, while those shown in red are related with a mesenchymal phenotype. Blue and red lines are used to indicate induction or repression, respectively.

Cell fate and phenotype are strictly regulated by extracellular signals. 1,25(OH)_2_D_3_ and EMT-TFs have opposite effects on epithelial cell phenotype and they antagonize each other (Figure 1). 1,25(OH)_2_D_3_ induces epithelial differentiation while it inhibits the expression of several EMT inducers. Conversely, expression of key EMT-TFs in epithelial cells promotes the acquisition of a mesenchymal phenotype, which in the case of SNAIL1 and SNAIL2 is associated with *VDR* gene repression and the blockade of 1,25(OH)_2_D_3_ action on epithelial differentiation. Thus, a double negative feedback loop operates between 1,25(OH)_2_D_3_ and EMT inducers that may contribute to the complete acquisition of the phenotype dictated by the extracellular cues. The loop may first amplify the signal and later stabilize cell fate once the process is completed. Hence, the balance between 1,25(OH)_2_D_3_/VDR and SNAIL family of transcription factors determines cell fate, and its imbalance may explain the reversibility of the EMT process. Of note, the transition between epithelial and mesenchymal phenotypes is also governed by similar double negative feedback loops among EMT-TFs and certain microRNAs, such as the ZEB/*miR-200* and the SNAIL1/*miR-34* regulatory circuits [22, 118–120].

The implication of EMT in cancer progression and organ fibrosis and the inhibitory effect of 1,25(OH)_2_D_3_ on EMT have opened the possibility of a therapeutic use of VDR agonists against these diseases. However, the downregulation of VDR expression found in several types of cancer, frequently associated with advanced stages of the disease, limits the applicability of vitamin D compounds to prevention in high-risk populations and treatment in patients at early stages of tumor progression. In addition, EMT is a transient event during tumorigenesis, and it has been proposed that the reverse process (MET) is required for the establishment of metastasis at distant sites [121, 122]. These lines of evidence have led to controversy about anticancer therapeutic strategies designed to inhibit EMT, as they may favor the formation of metastases, and suggest that these therapies may be limited to patients diagnosed at early stages of the disease to prevent invasion and dissemination [23]. Nevertheless, vitamin D compounds as inhibitors of EMT may be interesting therapeutic agents for fibrosis-associated pathologies, in which the EMT process is not reverted and the mesenchymal phenotype is maintained during disease progression.

## Figures and Tables

**Table 1:** List of 1,25(OH)_2_D_3_-regulated proteins involved in EMT.

Protein	1,25(OH)_2_D_3_ effect	Reference
Tight junction components		
Occludin	Upregulation	[26–28]
Claudin-1	Upregulation	[27, 29]
Claudin-2	Upregulation	[29, 30]
Claudin-7	Upregulation	[31]
Claudin-12	Upregulation	[30]
ZO-1	Upregulation	[26, 27, 29]
ZO-2	Upregulation	[26]
Adherens junction proteins		
E-cadherin	Upregulation	[26, 29, 31–46]
N-cadherin	Downregulation	[37, 41, 42, 47, 48]
P-cadherin	Downregulation	[37]
Vinculin	Upregulation	[26, 31]
Focal adhesion members		
Integrin *α*_3_	Upregulation	[31]
Integrin *α*_V_	Upregulation	[37]
Integrin *β*_5_	Upregulation	[37]
Integrin *α*_6_	Downregulation	[37]
Integrin *β*_4_	Downregulation	[37]
Paxillin	Upregulation	[31, 37]
FAK	Upregulation	[37]
Cytoskeleton-related proteins		
Filamin A	Upregulation	[49]
Ezrin	Upregulation	[50]
*α*-SMA	Downregulation	[37, 43–46, 51–54]
Keratin-13	Upregulation	[49]
Vimentin	Downregulation	[40–42]
Plectin	Upregulation	[49]
Extracellular matrix proteins		
Fibronectin	Downregulation	[44, 45, 51, 54]
Collagen type I	Downregulation	[44, 45, 51, 53–57]
Collagen type II	Downregulation	[56]
Collagen type III	Downregulation	[44, 51, 54, 58]
MMPs and inhibitors		
MMP2	Downregulation	[39, 41, 42]
MMP9	Downregulation	[39, 41, 42, 59, 60]
MMP13	Downregulation	[48, 61]
TIMP1	Upregulation	[59, 60]
TIMP2	Upregulation	[59]
EMT-TFs		
SNAIL1	Downregulation	[40–42, 44, 61, 62]
SNAIL2	Downregulation	[42, 61, 62]
ZEB1	Downregulation	[40]
TWIST1	Downregulation	[61]
Wnt/*β*-catenin target genes		
MYC	Downregulation	[26, 32, 63, 64]
TCF1	Downregulation	[26, 32]
CD44	Downregulation	[26, 32]
Cyclin D1	Downregulation	[31, 33, 55, 64]
AXIN2	Downregulation	[65]
LEF1	Downregulation	[65]
Other EMT-related proteins		
JMJD3	Upregulation	[66]
Cystatin D	Upregulation	[67]
Cathepsin L	Downregulation	[68]
Sprouty-2	Downregulation	[69]
PIT1	Downregulation	[70]
IL-1*β*	Downregulation	[71]
TGF-*β*	Downregulation	[44, 54, 56, 58]
TGF-*β* receptor type I	Downregulation	[44]
Cadherin-17	Downregulation	[72]
